# Scenarios for population health inequalities in 2030 in Europe: the EURO-HEALTHY project experience

**DOI:** 10.1186/s12939-019-1000-8

**Published:** 2019-06-25

**Authors:** António Alvarenga, Carlos A. Bana e Costa, Carme Borrell, Pedro Lopes Ferreira, Ângela Freitas, Liliana Freitas, Mónica D. Oliveira, Teresa C. Rodrigues, Paula Santana, Maria Lopes Santos, Ana C. L. Vieira

**Affiliations:** 1 ALVA Research and Consulting, Lisbon, Portugal; 2 CEG-IST, Centre for Management Studies of Instituto Superior Técnico, Universidade de Lisboa, Av. Rovisco Pais, 1049-001 Lisbon, Portugal; 3 IHC, Institute of Contemporary History, Universidade NOVA de Lisboa, Lisbon, Portugal; 4 Nova School of Business and Economics, Universidade NOVA de Lisboa, Campus de Carcavelos, Rua da Holanda 1, 2775-405 Carcavelos, Portugal; 5 ISEG, Lisbon School of Economics & Management, Universidade de Lisboa, Lisbon, Portugal; 6 Agència de Salut Pública de Barcelona, Barcelona, Spain; 7 CIBER of Epidemiology and Public Health (CIBERESP), Madrid, Spain; 8 Center for Health Studies and Research (CEISUC), University of Coimbra, Coimbra, Portugal; 9 Centre of Studies in Geography and Spatial Planning (CEGOT), University of Coimbra, Coimbra, Portugal

**Keywords:** Health inequalities, Population Health, Participatory approach, Delphi method, Foresight, Policies, Socio-technical approach, Scenarios, Stakeholders

## Abstract

**Background:**

Health inequalities have been consistently reported across and within European countries and continue to pose major challenges to policy-making. The development of scenarios regarding what could affect population health (PH) inequalities across Europe in the future is considered critical. Scenarios can help policy-makers prepare and better cope with fast evolving challenges.

**Objective:**

This paper describes the three 2030 time-horizon scenarios developed under the EURO-HEALTHY project, depicting the key factors that may affect the evolution of PH inequalities across European regions.

**Methods:**

A three-stage socio-technical approach was applied: i) identification of drivers (key factors expected to affect the evolution of PH inequalities across European regions until 2030) – this stage engaged in a Web-Delphi process a multidisciplinary panel of 51 experts and other stakeholders representing the different perspectives regarding PH inequalities; ii) generation of scenario structures – different drivers’ configurations (i.e. their hypotheses for evolution) were organized into coherent scenario structures using the Extreme-World Method; and iii) validation of scenario structures and generation of scenario narratives. Stages ii) and iii) were conducted in two workshops with a strategic group of 13 experts with a wide view about PH inequalities. The scenario narratives were elaborated with the participants’ insights from both the Web-Delphi process and the two workshops, together with the use of evidence (both current and future-oriented) on the different areas within the PH domain.

**Results:**

Three scenarios were developed for the evolution of PH inequalities in Europe until 2030: ‘*Failing Europe’* (worst-case but plausible picture of the future), ‘*Sustainable Prosperity’* (best-case but plausible picture of the future), and an interim scenario ‘*Being Stuck’* depicting a ‘to the best of our knowledge’ evolution. These scenarios show the extent to which a combination of Political, Economic, Social, Technological, Legal and Environmental drivers shape future health inequalities, providing information for European policy-makers to reflect upon whether and how to design robust policy solutions to tackle PH inequalities.

**Conclusions:**

The EURO-HEALTHY scenarios were designed to inform both policy design and appraisal. They broaden the scope, create awareness and generate insights regarding the evolution of PH inequalities across European regions.

## Background

Recognising the importance of health inequalities as a growing policy issue for the European Union (EU), the European Commission [1] issued some key recommendations to the Member States: “• lead on clear and comprehensive strategies to redress the current patterns and magnitude of health inequalities; • ensure the coherence and effectiveness of action to reduce health inequalities at all levels of government and across all sectors and stakeholders; • ensure that the capacities exist for coherent and effective implementation of action on health inequalities; • ensure progressive improvement in the availability and use of data needed to identify priorities, plan action, monitor trends and evaluate what actions are most effective” ([1], p. x). There are however several challenges in the design and implementation of policies for reducing health inequalities, not only on how to assist policy-makers to holistically evaluate policies’ benefits and to reflect upon their doability and power issues, but particularly on how to anticipate the extent to which future events may affect those policies [2, 3].

The field of foresight and scenario planning offers suitable tools to provide support and advice to policy-makers [4, 5] because the key to choose resilient (policy) actions is to define what they should be resilient to [6]. Looking at different plausible scenarios, decision-makers can anticipate possible or potential strategies [7] and use their knowledge to prepare for what may lie ahead, taking a proactive position instead of just accepting the events [8]. Applications of foresight methods to the health context has shown many advantages and offer insights to “be better equipped to improve health systems and interventions, and prepare for future public health incidents” ([9], p. 54). Vollmar and colleagues [10] reviewed 41 papers on the use of scenarios in the health field and concluded that, despite the great potential of scenarios as a strategic decision-making and healthcare planning tool, scenario building methods are not widely used when compared to other methods (e.g. consensus methods, simulation modelling). These authors also found that most published studies do not describe the scenario building process on a transparent and comprehensive way. The reported literature explored issues mainly related with diseases, public health on an organizational level, the healthcare labour market, technology, the pharmaceutical field and aging [10], but up to our knowledge no study has focused on population health (PH) inequalities across European regions.

The EURO-HEALTHY H2020 research project (with EURO-HEALTHY standing for ‘Shaping EUROpean policies to promote HEALTH equitY’) (2015-2017) [11] proposes a multicriteria Population Health Index (PHI) as a tool to help reflecting upon the future of PH inequalities and to assist policy evaluation. The development of the PHI was based on the definition of PH by Kindig and Stoddart [12], which acknowledges that accurate measurement of PH must consider the “health outcomes and their distribution within a population, the patterns of determinants that influence such outcomes, and the policies that influence the optimal balance of determinants” ([12], p. 382). Accordingly, the PHI has two components, one regarding health determinants and another regarding health outcomes [13]. Departing from this approach and assuming that scenarios may provide new evidence on possible future developments, the EURO-HEALTHY project took the construction of PH scenarios as a key challenge to inform the evaluation of policies in the context of health inequalities evolution.

A key challenge regarding the scenarios built under the EURO-HEALTHY project was that they needed to be designed in a transparent and replicable way that considered not only the evidence (both current and future-oriented evidence) on the different areas of concern within the domain of PH, but also the diversity of perspectives and values of the full panoply of stakeholders across Europe [14]. This is aligned with literature suggesting that for scenarios to be relevant, consistent and useful, the scenario building process should involve “people whose futures are being discussed are part of the scenario development process” ([8], p. 346). Also, health research has recognised the importance of participatory processes in recent years [15], with a growing understanding that participation of both researchers and stakeholders potentiates dialogue towards health improvements and ways of addressing health inequalities [16–18].

Specifically, this paper describes the EURO-HEALTHY PH scenarios whose focal issue was the future of PH inequalities across European regions in 2030. The proposed scenarios aim to support European policy-makers, and the scientific community, to reflect upon what can affect health and health inequalities across European regions in the future, so as to assist policy design and appraisal.

## Methods

### Overview of the socio-technical approach for scenario building

The goal of our study was to develop PH scenarios able to facilitate European policy-makers’ reflection upon what could affect PH inequalities across Europe. Given the wide range of plausible variations in health determinants’ inequalities (and on their causal factors) across European regions, an array of possible futures (i.e. scenarios) should be envisaged in order to explicitly consider contextual uncertainty during policy design and appraisal. There are several methodological approaches for building scenarios [19]. Our methodological choice was aligned with the Extreme-World Method [20], having in view the advantage of providing a practical and transparent way of establishing plausible boundaries within which the future of PH inequalities in Europe can unfold. These boundaries are defined by two extreme, yet still plausible, contrasted scenarios, covering both the more optimistic (best-case) and the more pessimistic (worst-case) perspectives.

**Fig. 1:**
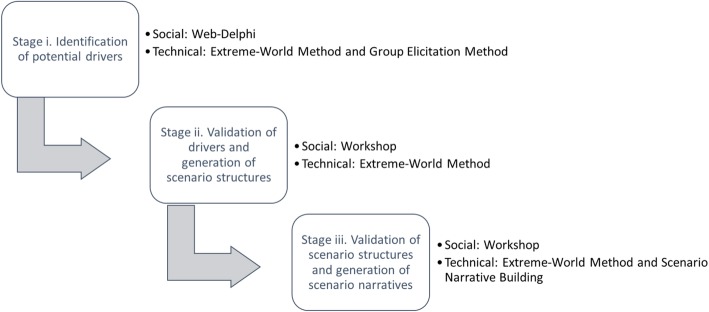
Three-stage socio-technical approach for scenario building

Specifically, the Extreme-World Method [20] was embedded into a three-stage scenario building socio-technical approach (see Fig. 1). *Stage i) ‘Identification of potential drivers’* aimed to determine the key factors that were expected to affect the evolution of PH inequalities across European regions until 2030 (these key factors are called *drivers* in the scenario terminology). The potential drivers were collected through a Web-Delphi process [21] implemented in the WELPHI Decision Support System [22] that was designed to drive a large multidisciplinary panel of experts and stakeholders, representing a wide range of perspectives regarding PH inequalities, into the process of identifying drivers relevant to the evolution of PH inequalities. The potential drivers were generated considering criteria adapted from the Group Elicitation Method (GEM) [23]. In its original application, GEM is a combination of the brain writing technique with a decision support system that allows to depart from an array of viewpoints and reformulate them in a list of concepts by applying different criteria – simplicity, interest, robustness and corroboration [23]. In our adaptation of GEM, the analysts set of number of criteria (see stage i) to then individually perform an individual analysis of the results of the first round of the Web-Delphi process.

*Stage ii) ‘Validation of drivers and generation of scenario structures’* was designed to create two contrasting scenario structures following the Extreme-World Method. For that purpose, a strategic group of 13 members – representing a wide range of perspectives regarding PH inequalities – participated in a workshop and validated the drivers collected in *stage i)* (along with the corresponding hypothesis for evolution, called *drivers’ configurations*) and further organized them into two scenario structures: one with all the worst-case (increasing PH inequalities) hypotheses of evolution, and the other with all the best-case (decreasing PH inequalities) hypotheses.

*Stage iii) ‘Validation of scenario structures and generation of scenario narratives’* kept the workshop format and aimed to adjust and validate the two contrasted scenario structures, as well as to build an interim scenario structure depicting the ‘to the best of our knowledge’ evolution, in view of giving rise to a reference scenario. The three scenario structures were validated and provided the backbones for the development of scenario narratives to enable a better understanding and communication of the scenarios, which were later enriched with future-oriented evidence that was collected.

Details from the implementation of each stage of the adopted socio-technical approach are described in the following sub-sections.

### Stage i) Identification of potential drivers

A total of 51 experts and other stakeholders were invited to take part of a Web-Delphi process to determine which key factors were expected to affect the evolution of PH inequalities across European regions until 2030 (the drivers). The participants included people from all over Europe, linked to the public and private sectors and the society at large. Their fields of expertise covered a large spectrum, such as public health, urban and regional planning, social inequalities, environment, and groups at-risk [14]. The Web-Delphi included two rounds: round 1 with open-ended questions for idea-generation regarding the reasons for possible evolutions in PH determinants in Europe; and round 2 for the participants to state their agreement regarding the potential drivers obtained in round 1.

In round 1, participants were faced with sets of health determinants’ indicators (i.e. integrating the health determinants component of the PHI), organized per PH area of concern (list of indicators within each area of concern provided in Table 2 in Appendix 1), together with their respective performance ranges across regions (reference year 2014; info with reference to the European NUTS 2 regions). For each area of concern, the participants were asked the ‘trigger-question’: *Given the set of current gaps across European regions on the [area of concern] indicators, shown on the left side of your screen, please indicate which of the following three statements do you consider to be plausible* (you can select more than one). The options presented (in text boxes) were the following: *Until 2030, there will be [an increase, a decrease and/or no change] in [area of concern] inequalities across European regions for the following main reasons*. Participants were then invited to opt for a statement of increase, decrease and/or no change on PH inequalities, and then to provide one or two main reasons for each of those selections. An illustrative example for the area of concern *Economic conditions, social protection and security* is provided in Figure 4 in Appendix 1. The participants’ answers provided a list of potential drivers generated taking into account the following criteria adapted from GEM [23]: (i) address a specific issue, (ii) be non-redundant, (iii) be simple, and (iv) be understandable.

In round 2, participants were presented with the list of potential drivers, organized into six PESTLE categories (Political, Economic, Social, Technological, Legal and Environmental). The PESTLE framework is recognised in the literature as an useful tool to identify key drivers of change in scenario building exercises [24]. For each potential driver, participants had to express their level of agreement regarding its relevance, reacting to the following statement: *By itself, this driver can originate a change in population health inequalities until 2030*. The answers were given in a five-level Likert scale (‘Strongly Disagree (SD)’, ‘Disagree (D)’, ‘Neither Agree nor Disagree (NAD)’, ‘Agree (A)’, ‘Strongly Agree (SA)’). Group agreement, which was meant to determine either approval or rejection of any given driver, was analysed by applying specific rules for dealing with differences in opinion. Two rules for approval were established to select drivers (see Table 3 in Appendix 1) and at least one driver from each PESTLE category was included.

### Stage ii) Validation of drivers and generation of scenario structures

A strategic group of 13 experts and stakeholders participated in a first (face-to-face) workshop that targeted the validation of drivers and the development of contrasted, extreme scenario structures. The 13 participants were divided in two groups that worked separately with a coherent sub-set of PESTLE categories and the respective drivers obtained by the end of Stage i). Each group started by discussing the drivers and their configurations – i.e. the worst-case (increasing PH inequalities) and best-case (decreasing PH inequalities) hypotheses of evolution. The discussion resulted in two preliminary scenario structures: one for the worst-case and one for the best-case. The scenario structures’ internal consistency was subsequently analysed, and redundancies were eliminated. This workshop ended with the members of the strategic group discussing the results obtained by each sub-group.

### Stage iii) Validation of scenario structures and generation of scenario narratives

The strategic group gathered in a second workshop to adjust and validate the two contrasted scenario structures, and to build an interim scenario structure depicting the ‘to the best of our knowledge’ evolution, to give rise to a reference scenario. The group discussed the three scenario structures, along with key characteristics for scenarios, such as compatibility, meaningfulness, representativeness and plausibility [19]. To improve the description of each driver configurations – i.e. worst-case (increasing PH inequalities) and best-case (decreasing PH inequalities) – future-oriented evidence was collected (the search protocol is listed in Table 5 in Appendix 2) and a file with all the information was systematized for each driver. This file can be made available from authors upon request. The final task of this stage was the generation of scenario narratives and the development of factsheets describing each one of the three EURO-HEALTHY PH scenarios. The scenario structures previously validated in the second workshop provided the backbones for these narratives, with the future-oriented evidence being also used to enrich each storyline.

## Results

**Fig. 2:**
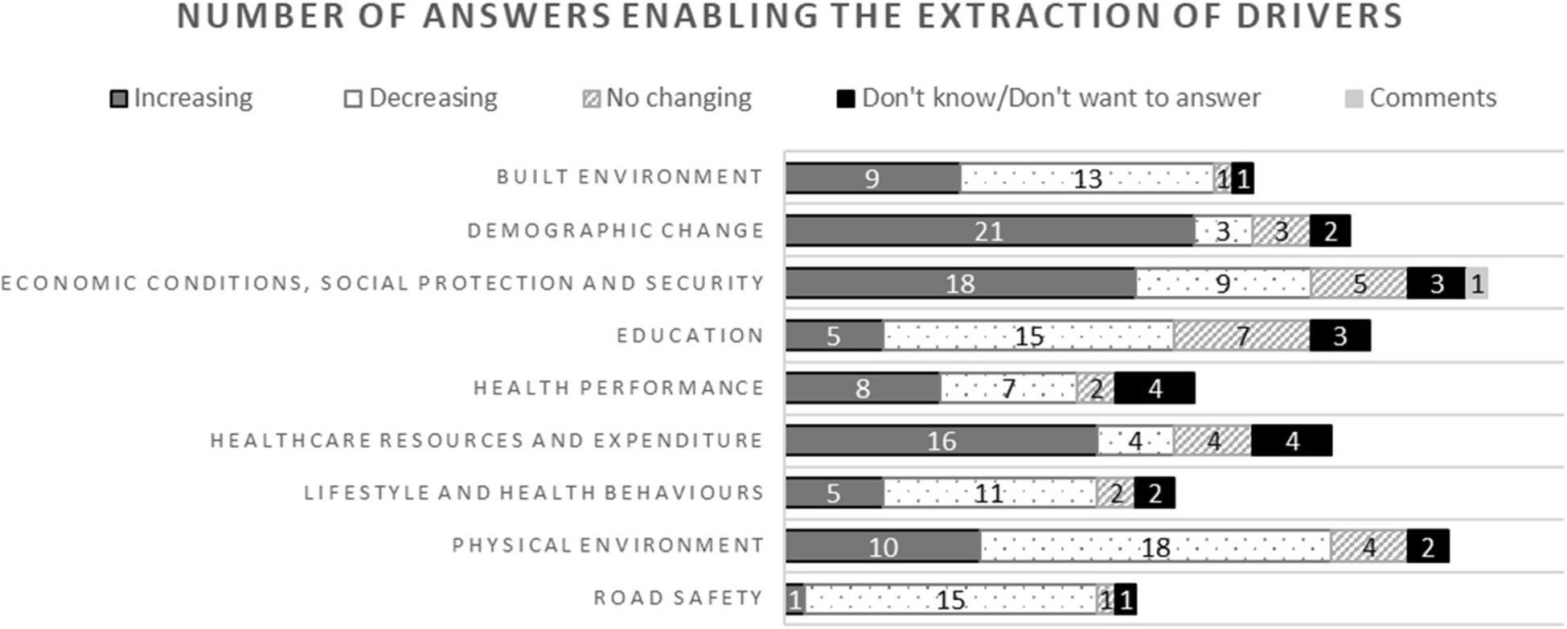
Number of answers used for extraction of drivers by PH area of concern and answer type

Analysing the results from the Web-Delphi round 1, five types of answers were identified: answers containing reasons for an increase in PH inequalities, for a decrease, and for no change; justifications for the ‘Don’t know/Don’t want to answer’ option; and additional comments left by participants during the process. These resulted in 362 answers collected, with 240 of these enabling the extraction of drivers – Fig. 2 presents the distribution of the 240 answers by area of concern and by type of answer.

The scrutiny of the 240 answers led to 178 drivers (see Table 4 in Appendix 1) that were included in the Web-Delphi round 2. The application of the selection rules defined (see Table 3 in Appendix 1) resulted in 49 drivers (see Table 4 in Appendix 1, drivers highlighted in bold). Then, the generation of scenario structures and the analysis of consistency during the workshops led to 36 drivers, which were plausible and coherent in the framework of the three scenario structures (stages ii) and iii) described above).

Table 1 presents the 36 drivers and corresponding configurations for each scenario structure, organised by PESTLE category: Political (seven drivers), Economic (10 drivers), Social (10 drivers), Technological (one driver), Environmental (seven drivers) and Legal (one driver). The worst-case scenario structure gathers the configurations that, once combined, describe a (plausible) increase in PH inequalities. On the opposite, the best-case scenario structure, gathers combinations leading to a (plausible) decrease in PH inequalities. The interim structure, defined as ‘to the best of our knowledge’ structure, contains the hypothesis that participants expect to happen given the current trends and the available information to this day.

**Table 1:** Overview of the three scenario structures, composed by drivers and drivers’ configurations

PESTLE category	Drivers	Worst-case (increase in inequalities)	To the best of our knowledge	Best-case (decrease in inequalities)
Political	Cohesion funds (or other funds) for less-favoured regions	Reduction	Maintaining	Maintaining
	Political commitment and public support towards universal access to healthcare	Weak	Weak	Strong
	Public expenditure in the healthcare system	Reductions	Priority with often insufficient funding	Increases
	Social protection policies for the elderly	Significantly weaker	Significantly weaker	Significantly stronger
	Investments in national social security systems	Significantly lower	Without significant change	Significantly higher
	Extent of compulsory education	Limited and non-harmonized	Extension with weak harmonization	Throughout the EU and increasingly harmonized
	Quality of Public Education	Decrease	A priority with often insufficient funding	Increase
Economic	Healthcare efficiency	Significant decrease	Without significant change	Significant increase
	Financial, Economic and Social crises	Deeper and long lasting	Occasional and regular	Mitigated cyclical global crises
	People's material deprivation	Increase	Small decrease	Significant decrease
	Economic inequalities	Increase	Increase	Decrease
	Social Insurance Schemes	Weakening	Without significant change	Strengthening
	Unemployment rate in Europe	Significantly higher	Without significant change	Significantly lower
	Long-term structural unemployment	Significant growth	Without significant change	Significant decrease
	Unemployment among 55+	Significant increase	Significant increase	Decrease
	Employment precariousness	Significant increase	Small increase	Decrease
	Employment with low income	Significant increase	Small increase	Decrease
Social	Concentration of people at of poverty and social exclusion	Higher	Higher	Lower
	Medical quality and effectiveness of healthcare services	Deterioration	Improvements with many dissimilarities	Significantly higher
	Access to healthcare	More limited	More limited	Widening
	Quality and accessibility of the primary healthcare services	Significant reduction	Maintaining	Steady growth
	Access and Quality of Emergency Medical Services (EMS) in remote and/or rural areas	Significantly lower	Without significant changes	Significantly higher
	Smoking restriction policies	Softer	Stricter	Stricter
	Diet and nutrition	Significantly less healthy	Limited improvement	Significantly more healthy
	Sedentary lifestyles	Increase	Limited reduction	Reduction
	Non-communicable diseases like diabetes and hypertension	Increase	Increase	Decrease
	Food security	Compromised	Without significant change	Improved
Technological	Medical innovation (improved and affordable medicines, medical research and technologies)	Stagnation	Slow growth	Rapid growth
Environmental	Quality of the built environment	Decrease	Without significant change	Significant improvement
	Quality of the natural environment	Decline	Without significant change	Improvement
	Quality of the outdoor air	Decline	Sustain the current levels	Improvement
	Climate change adaptation	Maladaptation	Limited adaptation	Improved resilient adaptation
	Climate change mitigation	Disengagement	Limited action	Engagement
	Priorities in terms of economic model: green-based vs. fossil fuel-based	Fossil fuel-based	Slow transition	Green-based
	Development and penetration of renewable energy production (water, wind, sun)	Slowdown	Increase	Significant and rapid increase
Legal	EU environmental policies and regulations (air, water, soil, waste, noise, chemicals)	Ineffective	Limited improvements in effectiveness	Effective

**Fig. 3:**
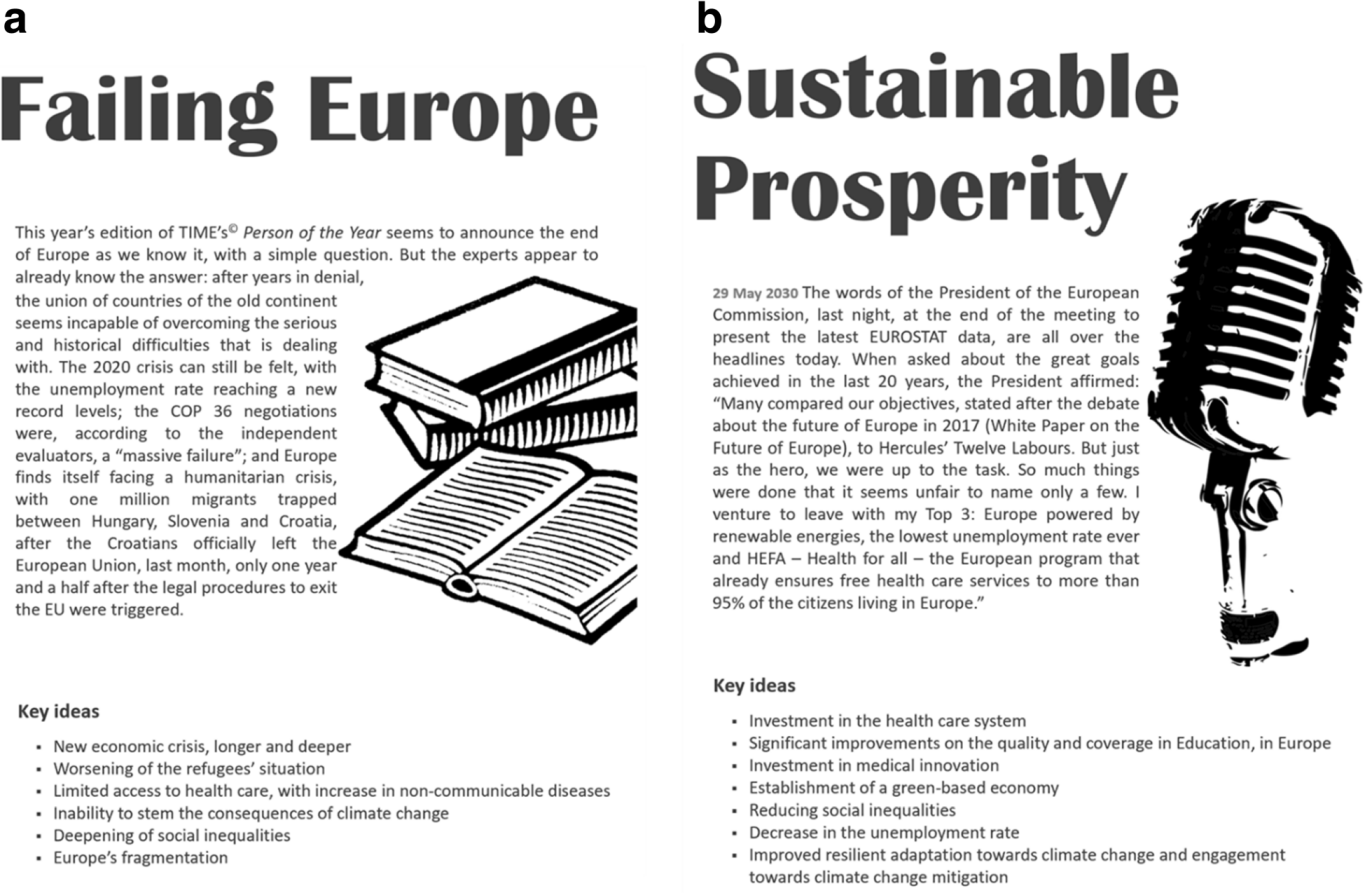
Factsheet for the ‘*Failing Europe*’ (**a**) and ‘*Sustainable Prosperity*’ (**b**) scenarios

**Fig. 4:**
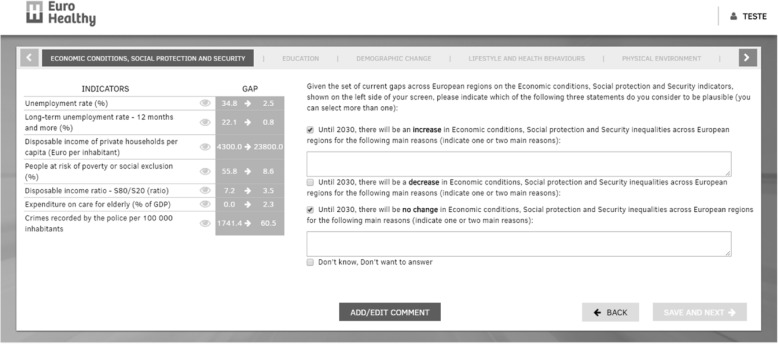
Print-screen showing the Web-Delphi round 1 questionnaire. Areas of concern of the EURO-HEALTHY PHI are on the top and the respective indicators are on the left. A text box opened when participants selected an option to answer

Out of these driver’s configurations, three scenarios were constructed and supported by a narrative and storyline. A short symbolic description containing a set of key ideas and the structure for the worst-case scenario ‘*Failing Europe*’ (Fig. 3a) and the best-case scenario ‘*Sustainable Prosperity*’ (Fig. 3b) were also developed as a result of the process.

## Discussion

### The EURO-HEALTHY scenario narratives

This paper presents the scenarios for the future of PH inequalities across European regions, for the 2030 time-horizon, developed under the EURO-HEALTHY project. Up to our knowledge these are the first PH scenarios that focus on PH inequalities across European regions, hence it is not possible to compare them with other scenario building exercises. However, it should be noted that (a) previous research has recognized there is a large number of determinants of inequalities in health [25], which is aligned with the EURO-HEALTHY scenarios’ complexity; and (b) some of the EURO-HEALTHY scenario drivers were also captured in four other large public health scenario studies [3, 26–28].

Two highly contrasted scenarios (‘*Failing Europe*’ and ‘*Sustainable Prosperity*’) and one reference scenario (‘*Being Stuck*’) are now outlined and discussed in the light of health inequalities in Europe in 2030. The ‘*Failing Europe*’ scenario – the worst-case scenario – assumes an increase in inequalities, whereas ‘*Sustainable Prosperity*’ corresponds to the best-case scenario structure, which includes all the configurations that lead to a decrease in PH inequalities. The reference scenario ‘*Being Stuck*’, corresponds to a narrative reflecting the “to best of our knowledge”. To comprehensively capture the discussion being conducted it is necessary that the reader of this paper positions her/himself in the future, as in the style of scenario literature. Furthermore, it is important that the reader bears in mind that the scenarios narratives were built departing from the scenario structures developed (the scenario backbones), together with the insights coming from both the Delphi participants (informed by current evidence on PH inequalities) and the strategic group discussions in the two workshops (informed by both current and future-oriented evidence). Our expectation is that both the scenario narratives content and the exercise of reading the scenarios while *being in the future* offer insights on key drivers of PH inequalities which are relevant to inform European health stakeholders and policymakers.

The text that follows describes what will be the future of PH inequalities across European regions, for the 2030 time-horizon, considering each one of the three EURO-HEALTHY scenarios and all the evidence gathered along the scenario building process.

#### ‘Failing Europe’ Scenario

In 2030, Europe plunged into a new, deeper, and long-lasting economic crisis. The already difficult situation is being amplified by the refugees’ situation, which it is not only far from being solved: it has deteriorated over recent years. The impacts on people’s lives are very negative with the increase of inequalities and limited access to basic services such as healthcare. With the social and economic challenges rising every day, other urgent issues such as tackling climate change, have been given second priority, exacerbating the already very serious impacts of the fragmented and ineffective laws and agreements that have been introduced over the last decade. Europe is no longer the solid, strong old continent that we were used to: connection and cohesion gave way to fragmentation.

Europe’s rough ride is very much linked to social and political decisions made over the past 15 years. The weak political commitment and consequent reduction of the funds available to support and help level the European economies are consider holding a significant part of the responsibility for bringing us to this unprecedented crisis. The expression *public investment* was wiped off the Europe leaders’ vocabulary. No one knows the exact moment people stopped being the priority, but the consequences are being felt. People’s material deprivation and social exclusion have augmented considerably. And the increasing number of financial and social barriers limits the access to basic services like healthcare services, which raises serious questions regarding, for instance, the increasing number of cases of non-communicable diseases. People are focused on making ends meet and, as some say, ‘cannot waste time and money’ on keeping healthy lifestyles.

The deeper consequences of an apparently silent enemy, climate change, are also unfolding at a faster pace. The government’s inaction towards climate changes, together with the ineffective EU environmental policies and regulations have caused, among others, a decrease in food security and availability, translating into an increase of basic food prices. The international disengagement towards climate change mitigation had other consequences, mainly related to the prevalence of a fossil fuel-based economic model: the green, alternative technologies needed today to try to overturn the effects of the maladaptation to climate changes do not exist because the necessary investments were not made at the right time. A new model needs to be settled as soon as possible in order to ensure minimal levels of quality of life for the citizens. This paradigm shift requires workforce to be fulfil. But, throughout Europe, the unemployment rates are significantly higher than 15 years ago, which means that the problem is not the quantity of available workforce. This is an emerging key dilemma of our times: the expertise needed to achieve the goal does not exist and the technology required to leverage and sustain the change in the economic paradigm does not exist or it is not sufficiently developed. Who is to blame? The lack of efforts made to improve and develop the quality of the public education and to harmonize and extent the public compulsory education and the slowdown of the development and penetration of renewable energy technologies might be part of the explanation. Europe stands with its hands tied behind its back. Is it too late now to say sorry?

#### ‘Sustainable Prosperity’ Scenario

It is no understatement to claim that Europe “is at its’ best”. Europe was able to mitigate the cyclical global crises, allowing a reduction of social inequalities in 2030. These important achievements were sustained by the maintenance of funds to help less-favoured regions and through investments in crucial areas such as healthcare and innovation. After the conclusions presented in the White Paper on the Future of Europe [29], in 2017, the European leaders gathered and decided that Europe needed to make partnerships with other economies and companies worldwide and adopt a forward-looking approach, with all the effort that would take: focusing on technological development and people’s quality of life allowed to establish a green-based economy and to decrease the unemployment rates. The technology progresses along with the implementation of effective EU environmental policies lead Europe to improve its adaptation towards climate change, “making peace” with both the planet Earth and the European citizens.

The European integration process is alive and delivering. As mentioned, the cyclical global crises were mitigated, and the economic inequalities decreased. The most remarkable and evident consequence of this economic prosperity is the significant decrease in people’s material deprivation and in people at risk of poverty, as well as the general improvement in the quality of life. Europe effectively solved the refugees’ crisis, something that seemed impossible 15 years ago. The concerns around the long-term sustainability of the healthcare systems and other public services did not block Europe’s determination to widen the access to healthcare nor the objective, fully accomplished, to significantly improve the medical quality and effectiveness of healthcare services. The goal to develop better public institutions involved a strong political commitment and implied the reinforcement of investments. The increase of public expenditure in the health system, for instance, was not only responsible for the decrease in non-communicable diseases, like diabetes and hypertension, but also contributed for significantly lower unemployment rates throughout Europe since the systems needed to be reinforced with qualified workforce. The decline of the unemployment rate was also influenced by the commitment to technology development, another goal set in the Europe’s bold view of the future. With that vision on one hand and the climate change challenges on the other, European countries made a joint effort towards the development of cutting-edge technologies, capable of enabling a successful transition from a fossil fuel-based paradigm to a Europe powered by renewable energies.

The results exceed the initial expectations: the new green economy, together with effective EU environmental policies and regulations helped mitigating climate change and supported the improvement of the quality of the natural environment, with direct consequences on the food security and on the population’s health and lifestyles in general. Europe has been sending a strong and clear message to the world: “old continent” is an outdated nickname. A statement included in the Schuman Declaration of 1950 [30], and recalled in the 2017 White Paper on the Future of Europe [29], can sum up Europe’s last decade very well: “Europe will not be made all at once, or according to a single plan. It will be built through concrete achievements which first create solidarity.”

#### ‘Being stuck’ Scenario

Europe finds itself in a crossroad. In 2030, the envisioned development and the transformation attempts continue to be held by political and economic conditions. The European countries failed to achieve their goals of speeding up the economic growth and create more and better jobs. Few things have changed in the last 15 years. People’s economic deprivation experienced a small decrease in general, but economic inequalities increased. People’s quality of life did not experience an improvement and old problems are still present problems. It was “now or never” for the European leaders to shape the future of Europe. Did Europe just miss its golden opportunity?

Europe’s current situation is the result of a sequence of failed or wasted intentions. After the 2008 economic crisis and the Brexit, Europe seemed to have learned a valuable lesson and the conditions were met to the start of a new era. But the reality turned out to be much more rough and harder to change than expected. One of Europe’s main challenges was to recover from the economic crisis and regain the citizens’ and the world confidence. But the economic growth was slow, and the unemployment rates had no significant changes. The creation of thousands of new jobs seemed feasible: the economic model needed to change and that was the perfect timing to invest and open space for all the unemployed workforce. But the green-model economy expansion was slow. Impacts were felt not only in the employment rates but also in the climate change adaptation process: it was limited and far from the objectives set after the Paris Agreement. Despite the governments’ engagement, leaders responded too slowly. EU environmental policies were ambitious and experienced a generic improvement in its effectiveness. But those improvements were limited by different interests and financial constraints. The development of renewable energies continued but always pressured by high completion from non-renewable sources and controlled by budget limitations. Investment was also a problem in other key areas. Insufficient investments in healthcare and social security prevailed, which limited the improvement of the efficiency of these systems; public expenditure in the healthcare system was a priority but the funding was insufficient to tackle all the needs.

Overall, we cannot clearly say that Europe is worse, or better, then 15 years ago. But postponing the resolution of so many major issues puts the old continent in a difficult position. Europe, nowadays (in 2030), is the embodiment of the well-known adage: ‘The road to hell is paved with good intentions’.

### Strengths and Limitations

The three-stage socio-technical approach adopted to develop the EURO-HEALTHY scenarios proved to be a transparent and meaningful approach, enabling a multidisciplinary and multidimensional understanding on how the future of PH in Europe may unfold. The combination of both the Web-Delphi process and the workshops allowed answering the technical challenges of identifying a list of drivers together with their hypotheses for evolution. The views and perspectives of a diverse and geographically dispersed group of experts, stakeholders and policy-makers are crucial in the process of scenarios building. In fact, this not only contributes for their validity but particularly meets the challenge of enhancing participation in scenario building. Furthermore, the scenarios proved to be plausible, relevant and showed a new and original perspective on PH inequalities. As an example, the two contrasted scenarios (best-case and worst-case) were applied to the EURO-HEALTHY Lisbon case-study, in which a panel of local stakeholders engaged in a participatory process of policy appraisal and prioritization of policies with potential to promote PH and decrease inequalities at the city level in light of the PH scenarios (more details can be found in [31]). The importance of having a reference scenario (the ‘to the best of our knowledge’ scenario) was also acknowledged given that in some policy contexts it may help people reflect on what is politically and economically realistic and most likely to occur, therefore boosting their critical reflection around the more extreme-case scenarios. As noted by Wack [32], a ‘business as usual’ scenario “build on the implicit views of the future shared by most managers, making it possible for them to recognize their outlook in the scenario package.” (p. 71). Our reference ‘to the best of our knowledge’ scenario, although different from a classic ‘business as usual’ scenario – because it acknowledges expected change (i.e. it does not imply a simple continuation of past trends and structural stability) – shares with the ‘business as usual’ conception the self-recognition quality highlighted by Wack. Of course, this can only happen when the reference scenario is framed, in the scenario building process, by other contrasted plausible scenarios, as it is the case in both our project and in the classic Shell case, avoiding the dangers of a single future consideration, as also reported by Wack [33].

There are limitations in the process of developing scenarios that should be considered and that go beyond the time-consuming and work-intensity issues. In addition to a subjective assessment and evaluation of complex facts, the type of scenarios depends greatly on the information basis provided and the imagination and background of the experts and other parties involved [34].

## Conclusions

The EURO-HEALTHY scenarios provide information on the most likely future development of health inequalities in Europe in light of the identified drivers. These follow the model structure designed within the PHI framework, defined *a priori* in 2015. New realities posing challenges to European cohesion, such as the growing mistrust on EU institutions, the cultural conflict over the refugee crisis and migrant influx, the rise of populist movements and terrorism, were not explicitly identified as key drivers affecting the future of health inequalities. Nevertheless, greater attention should be paid to these aspects, linked to the present social and political context, as they potentially affect the drivers selected in this study.

The scenario building process proved to be a transparent and replicable approach to produce substantively meaningful scenarios, with the participants in the process recognising its value and validating the scenarios developed. Hence, the scenarios obtained can be considered as a tool for European policy makers to have a better understanding about plausible future developments of PH, preparing them to counteract increases in inequalities.

## Data Availability

All data generated and/or analysed during this study are included in this published article and its additional files. The datasets used and/or analysed during the current study are available from the corresponding author on reasonable request. Anonymised fields will be used to ensure that individual participants are not identified.

## Appendix 1

### Identification of drivers

**Table 2:** Indicators used in Web-Delphi round 1, by area of concern of the EURO-HEALTHY Population Health Index

Area of concern	Indicator
Economic conditions, social protection and security	Unemployment rate (%)
	Long-term unemployment rate – 12 months and more (%)
	Disposable income of private households per capita (Euro per inhabitant)
	People at risk of poverty or social exclusion (%)
	Disposable income ratio – S80/S20 (ratio)
	Expenditure on care for elderly (% of GDP)
	Crimes recorded by the police per 100 000 inhabitants
Education	Population aged 25-64 with upper secondary or tertiary education attainment (%)
	Early leavers from education and training (%)
Demographic change	At risk of poverty rate of older people – aged 65 years or over (%)
	Ageing index (ratio)
Lifestyle and Health behaviours	Adults who are obese (%)
	Daily smokers – aged 15 and over (%)
	Pure alcohol consumption – aged 15 and over (Liters per capita)
	Live births by mothers under age of 20 (%)
Physical environment	Annual mean of the daily PM2.5 concentrations (μg/m^3^)
	Annual mean of the daily PM10 concentrations (μg/m^3^)
	Greenhouse Gas (GHG), total tonnes of CO_2_ eq. emissions per annum per capita
	Population exposed to traffic noise – Lden 55-59db, during day (%)
	Population affected by flooding, per 1 000 000 inhabitants
Built environment	Average number of rooms per person
	Households without indoor flushing toilet (%)
	Households without central heating (%)
	Population density (inhabitants/km^2^)
	Population connected to public water supply (%)
	Population connected to wastewater treatment plants (%)
	Recycling rate of municipal waste (%)
Road safety	Victims in road accidents – injured and killed, per 100 000 inhabitants
	Fatality rate due to road traffic accidents, per 1000 victims
Healthcare resources and expenditure	Medical doctors, per 100 000 inhabitants
	Health personnel (nurses and midwives, dentists, pharmacists and physiotherapists), per 100 000 inhabitants
	Total health expenditure (THE), PPP$ per capita, World Health Organization (WHO) estimates
	Private households’ out-of-pocket on health as percentage of total health expenditure (THE)
	Public expenditure on health, PPP$ per capita, WHO estimates
Healthcare performance	Hospital discharges due to diabetes, hypertension and asthma, per 100 000 inhabitants
	Amenable deaths to health care – standardised death rate, per 100 000 inhabitants

Source: Santana P, Costa C, Freitas A, Stefanik I et al. (2017) *Atlas of population health in European Union regions* (Imprensa da Universidade de Coimbra: Coimbra).

**Table 3:** Selection rules applied to the answers of the Web-Delphi round 2 considering the five-level Likert scale: ‘Strongly Disagree (SD)’, ‘Disagree (D)’, ‘Neither Agree nor Disagree (NAD)’, ‘Agree (A)’, ‘Strongly Agree (SA)’

Selection rules	Selection rule
First rule – applied to all drivers from all PESTLE categories	(SA + A) ≥ $\frac{2}{3}$ and SA ≥ $\frac{1}{3}$ and (SD + D) < 10%
Second rule – applied to drivers within the PESTLE categories in which the first rule did not select any driver	[(SA + A) ≥ $\frac{3}{4}$ or SA ≥ $\frac{1}{4}$)] and (SD + D) < 10%

**Table 4:** List of 178 potential drivers included in round 2 of the Web-Delphi, and distribution of the answers in that round

PESTLE category	Driver	SD	D	NAD	A	SA	DK/ DWtA
Political	Strength of liberal political orientations	7%	7%	10%	33%	38%	5%
Political	Capacity to produce EU agreements		7%	40%	48%	5%	
Political	EU integration process		2%	26%	55%	17%	
Political	Confidence in EU policies	2%	2%	43%	48%	5%	
Political	**Cohesion funds (or other funds) for less-favoured regions**		2%	7%	43%	48%	
Political	**Political commitment and ideological support towards universal access to healthcare**	2%	5%	10%	29%	55%	
Political	**State involvement in public health**	2%	2%	12%	33%	50%	
Political	Commitment to appropriateness of the healthcare systems in the EU		5%	31%	36%	26%	2%
Political	Market oriented reforms in healthcare (including privatization of healthcare)	7%	10%	14%	31%	38%	
Political	Priority for investment in innovation in health	5%	7%	14%	45%	29%	
Political	**Social protection policies for the elderly**		7%	5%	40%	48%	
Political	**Investments in national social security systems**	2%		10%	48%	40%	
Political	Global political evolution (e.g. the aftermath of the United States elections; Syria war, etc.)	2%	12%	26%	29%	31%	
Political	Power of populistic, protectionist and nationalist radical political projects in the EU	5%	7%	19%	29%	38%	2%
Political	Political stability in the EU and its Member-States (e.g. Brexit)	2%	7%	21%	50%	17%	2%
Political	Municipalisation [i.e. power to the municipalities]	5%	10%	33%	33%	17%	2%
Political	Application of road regulations/rules		10%	31%	48%	2%	10%
Political	Degree of penalization associated with traffic policies to reduce traffic accidents	2%	12%	31%	38%	7%	10%
Political	Policies for reducing traffic accidents in Eastern countries			29%	48%	19%	5%
Political	Levels of investment in road safety		5%	26%	48%	19%	2%
Political	Austerity policies	7%	5%	10%	31%	43%	5%
Political	**Growth of compulsory education**		5%	7%	40%	45%	2%
Political	Privatization of education	10%	17%	19%	31%	21%	2%
Political	**Investment in the modernization of the built environment**		5%	21%	40%	33%	
Economic	Financing of the healthcare sector (in proportion of the Gross Domestic Product) in more economically vulnerable countries	2%	2%	10%	55%	29%	2%
Economic	**Social insurance schemes**	2%	7%	10%	40%	40%	
Economic	Financial pressure on healthcare systems	5%	7%	10%	50%	29%	
Economic	**Public expenditure in the healthcare system**		2%	7%	50%	40%	
Economic	**Healthcare costs**	2%	5%	17%	36%	40%	
Economic	Constraints on fiscal policies		2%	33%	43%	14%	7%
Economic	Economic evolution in EU countries with a younger population compared to the economic evolution in EU countries with an older population		2%	24%	55%	12%	7%
Economic	Economic growth of some EU countries, namely of the ones from the former Eastern block	2%		24%	64%	10%	
Economic	Evolution of socioeconomic conditions and wealth in less developed countries/areas in Europe		2%	7%	57%	31%	2%
Economic	Economic evolution (e.g. Gross Domestic Product, productivity)		5%	24%	43%	26%	2%
Economic	Economic evolution in some countries of the Southern Europe (compared to the other EU countries)		2%	17%	52%	26%	2%
Economic	**Economic and social crises**			5%	43%	52%	
Economic	**Financial crisis**		2%	10%	38%	50%	
Economic	**Concentration of capital accumulation**	2%	5%	24%	36%	33%	
Economic	**People's economic stress**		2%	14%	38%	45%	
Economic	**Economic inequalities**	2%		2%	21%	74%	
Economic	Early retirement in some countries of the Southern Europe (compared to the other EU countries)		12%	52%	26%	7%	2%
Economic	Pension levels and differences in working after retirement		2%	19%	57%	21%	
Economic	Proportion of economically dependent population		2%	21%	45%	31%	
Economic	Evolution of financial burden share between generations within families		5%	26%	45%	24%	
Economic	**Financial pressures leading to cuts in social expenditures (e.g. pensions)**		2%	7%	40%	50%	
Economic	Differences in pension regimes within the EU (e.g. differences in national net pension replacement rates)		2%	29%	45%	21%	2%
Economic	**Poverty levels**			7%	21%	71%	
Economic	**Social benefits**			12%	43%	45%	
Economic	Global economic integration and interrelations		7%	21%	45%	21%	5%
Economic	Inclusion of the refugees on the labour market	2%	10%	21%	40%	26%	
Economic	**Unemployment rate in Europe**		2%	2%	48%	48%	
Economic	**Long-term structural unemployment**			2%	33%	64%	
Economic	**Unemployment in late ages**			7%	43%	50%	
Economic	**Employment precariousness**			2%	52%	43%	2%
Economic	**Employment with low income/limited social benefits**			7%	43%	50%	
Economic	Unemployment in countries of the Southern Europe (compared to the other EU countries)		7%	21%	43%	24%	5%
Economic	Employment opportunities for people with low qualifications		2%	17%	64%	17%	
Economic	Employment opportunities for medical/health personnel	2%	14%	29%	45%	10%	
Economic	Disparities in industry production		14%	50%	24%	7%	5%
Economic	Investment in formal education		2%	24%	45%	29%	
Economic	Demand for qualified workers		10%	33%	48%	7%	2%
Social	Refugees and migrants’ fluxes		7%	17%	60%	14%	2%
Social	Migrations to the most economically developed North-West European countries		7%	26%	57%	7%	2%
Social	Emigration from EU countries already affected by population ageing and decrease in natality		12%	26%	48%	12%	2%
Social	Urbanisation (attractiveness of urban areas)		7%	31%	50%	12%	
Social	Concentration of people in suburban areas		7%	33%	45%	14%	
Social	**Concentration of people at risk (e.g. poverty) in problematic neighbourhoods**		2%	7%	48%	43%	
Social	Differential in regions’ ability (political and/or economic) to attract young people, including migrants and refugees		5%	21%	55%	17%	2%
Social	Population ageing		14%	19%	31%	36%	
Social	Quantitative evolution of the young population		14%	36%	36%	10%	5%
Social	Birth rates across EU countries		14%	43%	33%	10%	
Social	Birth rates in some countries of the Southern Europe (compared to the other EU countries)		14%	45%	33%	7%	
Social	Ageing pace in Central and Eastern Europe compared to other EU countries		12%	43%	40%	5%	
Social	Regional differences in ageing patterns at a European level		12%	40%	40%	7%	
Social	Life expectancy		12%	19%	43%	21%	5%
Social	Healthy life expectancy		10%	17%	29%	38%	7%
Social	Differential in regions’ health expectancy		7%	19%	50%	17%	7%
Social	Voting power of the elderly population	2%	12%	60%	21%	5%	
Social	Population growth in relation to the “housing capacity”		7%	55%	29%	5%	5%
Social	Awareness of the road safety problematic		17%	45%	31%	5%	2%
Social	Road safety campaigns and education	2%	10%	38%	40%	7%	2%
Social	Social pressure for “bridging the gap” in terms of built environment		5%	29%	62%	5%	
Social	Overall quality of road infrastructure	2%	2%	31%	55%	7%	2%
Social	Quality of the roads in Southern and Eastern Europe	2%	7%	29%	52%	7%	2%
Social	Vehicles quality and safety		2%	36%	50%	10%	2%
Social	Metropolization		2%	45%	45%	5%	2%
Social	Traffic density		5%	38%	38%	19%	
Social	**Disparities in medical standards**		2%	17%	43%	36%	2%
Social	**Medical quality**		5%	17%	38%	40%	
Social	**Medical effectiveness of healthcare services**		2%	12%	45%	40%	
Social	**Access to healthcare**			5%	33%	62%	
Social	**Access to hospital emergency room**		5%	10%	52%	33%	
Social	**Quality and accessibility of the primary health care services**			5%	38%	57%	
Social	**Access to healthcare services in rural areas**			10%	40%	48%	2%
Social	**Quality of Emergency Medical Services (EMS) in remote and/or rural areas**		2%	10%	50%	36%	2%
Social	Emergency Medical Services efficiency		5%	19%	45%	31%	
Social	European medical network		7%	36%	45%	12%	
Social	European citizens’ health literacy and collaboration capacity as patients			29%	43%	29%	
Social	Educational and public health campaigns		10%	21%	43%	26%	
Social	Education and training of health professionals		10%	24%	43%	24%	
Social	Mobility of health professionals between countries and regions		10%	36%	40%	12%	2%
Social	Concentration of equipment and clusters of medical activities		5%	43%	36%	17%	
Social	Availability of health infrastructures		2%	19%	48%	31%	
Social	**Coverage of National Health Service**			5%	52%	40%	2%
Social	Response capacity of the health sector (quality of emergency/first respondents and hospital care) in less developed countries			12%	55%	31%	2%
Social	**Quality of healthcare organization and management**		5%	10%	48%	38%	
Social	Knowledge sharing		2%	33%	36%	26%	2%
Social	Availability of information on better lifestyles		5%	36%	36%	24%	
Social	Quality of the education in the EU			17%	52%	31%	
Social	Academisation of the society		7%	40%	40%	10%	2%
Social	Low qualifications in the EU			29%	64%	7%	
Social	Impacts on the quality of jobs and enthusiasm for Tertiary Education			38%	52%	5%	5%
Social	Monitoring of education results		7%	55%	36%	2%	
Social	Capacity of Eastern European countries to cope with the collapse of the soviet society [i.e. with the loss of some of its key features, for instance in education]		10%	45%	38%	5%	2%
Social	Educational attainment as an aspiration of EU countries		10%	29%	50%	7%	5%
Social	Objectives in education in the EU		5%	43%	36%	14%	2%
Social	Levels of education and literacy		2%	2%	69%	24%	2%
Social	Flexibility of academic curricula in the EU	2%	12%	48%	31%	2%	5%
Social	University fees		7%	40%	36%	12%	5%
Social	Academisation of job profiles		14%	50%	29%	2%	5%
Social	Existence of vocational training		10%	36%	43%	7%	5%
Social	Differences in cultural traditions		19%	31%	36%	10%	5%
Social	Globalization of style life		17%	31%	43%	5%	5%
Social	Awareness regarding the impact of lifestyles on quality of life		14%	17%	50%	14%	5%
Social	**Stress**		7%	17%	33%	40%	2%
Social	Awareness campaigns tackling alcohol consumption		12%	12%	52%	24%	
Social	Awareness regarding the impact of smoking		10%	14%	48%	29%	
Social	**Smoking restriction policies**		2%	2%	48%	48%	
Social	**Smoking among women**			12%	40%	48%	
Social	Smoking among manual workers			14%	52%	31%	2%
Social	Taxation and control of unhealthy food consumption		2%	10%	57%	31%	
Social	**Diet**		5%	17%	38%	40%	
Social	Disparities in health behaviours		7%	10%	52%	31%	
Social	**Sedentary lifestyles**			17%	43%	40%	
Social	**Evolution of Non-communicable diseases like diabetes and hypertension**		2%	10%	43%	43%	2%
Social	Social mobility (change in hierarchies in socio-economic positions)		7%	14%	52%	26%	
Social	Motorization in Eastern countries		7%	45%	38%	5%	5%
Technological	***Medical innovation (new medicines and technologies)***		5%	17%	45%	31%	2%
Technological	Technology and medical devices at home		10%	19%	45%	24%	2%
Technological	***Technology and innovation costs in healthcare systems***		7%	17%	50%	26%	
Technological	Evolution of vehicles batteries’ autonomy		19%	40%	31%	10%	
Technological	Knowledge and innovation in technology and materials for road safety		10%	33%	45%	7%	5%
Technological	Innovation in technology and materials for the built environment		10%	33%	40%	14%	2%
Technological	Innovation in housing (new forms of housing)		14%	33%	33%	14%	5%
Technological	Price of technology and materials for road safety and for the built environment		7%	38%	38%	14%	2%
Technological	Acceleration in industry development in less developed countries		14%	33%	38%	12%	2%
Technological	Pace of change in manufacturing in Europe (industry 4.0)		14%	43%	26%	12%	5%
Technological	Automation of industrial production		7%	40%	33%	14%	5%
Technological	Internet access	2%	5%	33%	48%	12%	
Environmental	**Quality of the living environment**		2%	2%	50%	45%	
Environmental	**Quality of the outdoor air**		2%		38%	60%	
Environmental	**Climate change**		2%	14%	43%	38%	2%
Environmental	**Climate change actions and policies**		5%	17%	43%	33%	2%
Environmental	Concerns related to natural resources efficiency (e.g. energy efficiency)		7%	26%	43%	24%	
Environmental	Energy efficiency efforts		10%	19%	52%	19%	
Environmental	Recycling: taxation and quotas		12%	29%	45%	14%	
Environmental	Social and political awareness/preoccupation with the physical environment, namely air and noise conditions		7%	14%	55%	24%	
Environmental	Gap in “environmental awareness” between more developed and less developed countries		2%	24%	48%	26%	
Environmental	**Priorities in terms of economic model: green-based vs oil-based (e.g. evolution of the number of environmental friendly vehicles)**		5%	10%	45%	36%	5%
Environmental	**Development of sustainable energy production (water, wind, sun)**			21%	38%	40%	
Environmental	Industrial development sustainability	2%	7%	29%	38%	24%	
Environmental	Regional differences of the building construction boom		14%	29%	52%	5%	
Environmental	Coordination of environmental-focused political measures		7%	21%	57%	14%	
Environmental	Environmental protection policies focused on industrial manufacturing		7%	17%	57%	17%	2%
Environmental	Environmental protection policies focused on vehicles circulation		5%	14%	60%	19%	2%
Environmental	International and worldwide environmental agreements		7%	14%	45%	31%	2%
Environmental	United States of America commitment towards the Paris Agreement on climate change global action		10%	14%	48%	26%	2%
Legal	EU Legislation/ Directives and its transposition into the legislation of each country		10%	33%	43%	10%	5%
Legal	Adaptability of EU regulations (especially inside the Eurozone) to Member-states specificities		7%	45%	33%	12%	2%
Legal	Commitment to follow EU directives and World Health Organization guidelines		5%	19%	52%	21%	2%
Legal	EU legislation / directives on built environment		7%	21%	57%	10%	5%
Legal	National building construction rules and legislation		12%	31%	40%	10%	7%
Legal	Differences in road safety national legislation	2%	7%	29%	55%	2%	5%
Legal	***EU environmental policies and regulations***		5%	17%	64%	12%	2%
Legal	Requirements of compliance to environmental regulations for market purposes (e.g. quality of fuel)		2%	24%	55%	17%	2%
Legal	Evolution of intellectual property rights in health	2%	12%	43%	24%	14%	5%
Legal	Impact of the TTIP (Transatlantic Trade and Investment Partnership) project in the access to the latest advances in medicines, diagnostic tools, and other life-saving medical technologies	5%	5%	45%	26%	17%	2%
Legal	Integration of EU labour markets		10%	38%	38%	10%	5%

*SD* Strongly Disagree, *D* Disagree, *NAD* Neither Agree nor Disagree, *A* Agree, *SA* Strongly Agree, *DK/DWtA* Don’t Know/Don’t Want to Answer

Note: the 49 drivers selected after round 2, by applying the selection rules, are highlighted in **bold**; among these, in ***italic***, are the drivers that resulted from the application of the second selection rule.

## Appendix 2

### Search protocol to collect future-oriented evidence

**Table 5:** Search protocol used to collect future-oriented evidence to inform the scenario building process (March of 2017)

Sources	European Union (EU) publications, through the EU Bookshop | https://bookshop.europa.eu/en/home FRESHER project | https://www.foresight-fresher.eu/fresher-project-results/
Keywords	Search words combined with drivers’ keywords (combinations followed this order): future; forecast; scenario; foresight; prospective [If the search did not deliver meaningful results, the protocol was extended to searches with Google’s Search Engine, using the driver’s keywords (or equivalent) + the expression *European Union*. Search results obtained beyond the Search Protocol were pointed out in the drivers’ cards]
Timespan	2012-2017
Language	English

## Abbreviations

EU: European Union
EURO-HEALTHY: Shaping EUROpean policies to promote HEALTH equitY
PESTLE: Political, Economic, Social, Technological, Legal and Environmental
PH: Population Health
PHI: Population Health Index
WHO: World Health Organization
